# Crystal structure of a mixed-valence μ-oxide Sn_12_ cluster

**DOI:** 10.1107/S1600536814023460

**Published:** 2014-10-29

**Authors:** Marina M. Kireenko, Kirill V. Zaitsev, Sergey S. Karlov, Mikhail P. Egorov, Andrei V. Churakov

**Affiliations:** a Department of Chemistry, M.V. Lomonosov Moscow State University, Leninskie Gory 1/3, Moscow 119991, Russian Federation; b N.D. Zelinsky Institute of Organic Chemistry, Leninsky Prospekt 47, Moscow 119991, Russian Federation; c Institute of General and Inorganic Chemistry, Russian Academy of Sciences, Leninskii Prospekt 31, Moscow 119991, Russian Federation

**Keywords:** crystal structure, stannylenes, S_12_ cluster, carbonyls

## Abstract

The mixed-valence μ-oxide Sn_12_ cluster, deca­carbonyl­tetra-μ_4_-oxido-hexa-μ_3_-oxido-tetra­kis­[μ-2,2′-(pyridine-2,6-di­yl)bis(1,1-di­phenyl­ethano­lato)]deca­tin(II)ditin(IV)dimolyb­denum(O)(2 *Mo*—*Sn*) toluene hepta­solvate, [Mo_2_Sn_12_(C_33_H_27_NO_2_)_4_O_10_(CO)_10_]·7C_7_H_8_, has a crystallographically imposed inversion centre. The asymmetric unit also contains three and a half toluene solvent mol­ecules, one of which is disordered about a centre of symmetry. The complex mol­ecule comprises six distinct Sn atom species with four different coordination numbers, namely 3, 4, 5, and 6. The Sn^II^ atoms forming the central Sn_10_O_10_ core adopt distorted trigonal–pyramidal, square-pyramidal and octa­hedral coordination geometries provided by the μ-oxide atoms and by the O- and N-donor atoms of two pyridinedi­ethano­late ligands. The terminal Sn^IV^ atoms have distorted trigonal–bipyramidal coordination geometries, with a μ_4_-oxide atom and the N atom of a pyridinedi­ethano­late ligand occupying the axial positions, and the Mo atom of a Mo(CO)_5_ group and the alk­oxy O atoms of a ligand forming the equatorial plane. In the crystal, weak intra- and inter­molecular C—H⋯O hydrogen bonds are observed.

## Related literature   

For general background to the chemistry of stannylene complexes with transition metals, see: Baumgartner & Marschner (2014 ▶); Lee & Sekiguchi (2010 ▶). For our previous work on heavy carbene analogs, see: Kireenko *et al.* (2012 ▶, 2013 ▶); Huang *et al.* (2012 ▶, 2013 ▶).
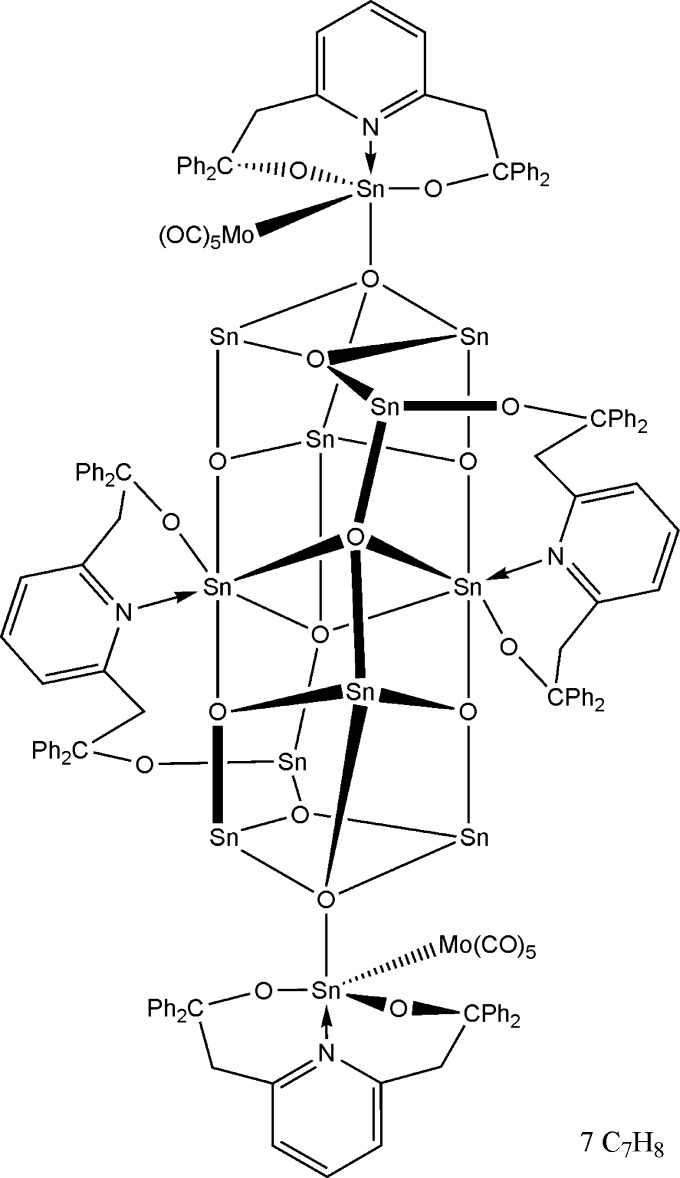


## Experimental   

### Crystal data  

[Mo_2_Sn_12_(C_33_H_27_NO_2_)_4_O_10_(CO)_10_]·7C_7_H_8_
*M* *_r_* = 4579.42
Triclinic, 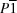

*a* = 15.8218 (17) Å
*b* = 15.9933 (17) Å
*c* = 18.806 (2) Å
α = 94.833 (2)°
β = 95.014 (2)°
γ = 110.605 (2)°
*V* = 4403.5 (8) Å^3^
*Z* = 1
Mo *K*α radiation
μ = 1.88 mm^−1^
*T* = 173 K
0.25 × 0.10 × 0.08 mm

### Data collection  

Bruker SMART APEXII diffractometer
Absorption correction: multi-scan (*SADABS*; Bruker, 2008 ▶) *T*_min_ = 0.651, *T*_max_ = 0.864
32165 measured reflections
15393 independent reflections
12647 reflections with *I* > 2σ(*I*)
*R*_int_ = 0.028

### Refinement  

*R*[*F* ^2^ > 2σ(*F* ^2^)] = 0.033
*wR*(*F* ^2^) = 0.081
*S* = 1.02
15393 reflections
939 parameters
13 restraints
H-atom parameters constrained
Δρ_max_ = 1.05 e Å^−3^
Δρ_min_ = −0.86 e Å^−3^

### 

Data collection: *APEX2* (Bruker, 2008 ▶); cell refinement: *SAINT* (Bruker, 2008 ▶); data reduction: *SAINT*; program(s) used to solve structure: *SHELXTL* (Sheldrick, 2008 ▶); program(s) used to refine structure: *SHELXTL*; molecular graphics: *SHELXTL*; software used to prepare material for publication: *SHELXTL*.

## Supplementary Material

Crystal structure: contains datablock(s) I. DOI: 10.1107/S1600536814023460/rz5135sup1.cif

**Fig. 1.**
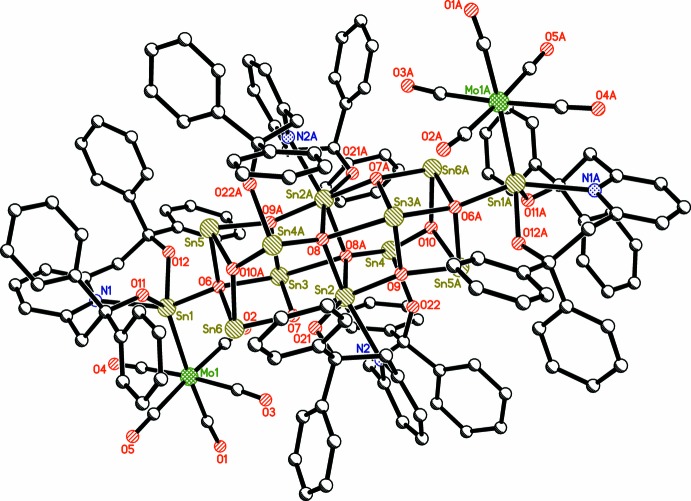
The molecular structure of the title compound, with displacement ellipsoids shown at the 50% probability level. Toluene solvent molecules, hydrogen atoms and labels for carbon atoms are omitted for clarity. Suffix A indicates the symmetry operator 2-x, 2-y, -z.

Structure factors: contains datablock(s) I. DOI: 10.1107/S1600536814023460/rz5135Isup2.hkl

x y z . DOI: 10.1107/S1600536814023460/rz5135fig1.tif
The mol­ecular structure of the title compound, with displacement ellipsoids shown at the 50% probability level. Toluene solvent mol­ecules, hydrogen atoms and labels for carbon atoms are omitted for clarity. Suffix A indicates the symmetry operator 2-*x*, 2-*y*, −*z*.

CCDC reference: 1030879

Additional supporting information:  crystallographic information; 3D view; checkCIF report

## Figures and Tables

**Table 1:** Hydrogen-bond geometry (, )

*D*H*A*	*D*H	H*A*	*D* *A*	*D*H*A*
C22H22*A*O9	0.99	2.28	3.052(5)	134
C28H28*A*O7	0.99	2.48	3.378(5)	151
C33H33O1^i^	0.95	2.53	3.205(6)	128

Symmetry code: (i) 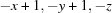.

## supplementary crystallographic information

### S1. Comment

Nowadays, carbenes and their heavy analogues (germylenes and stannylenes) are regarded as a new perspective ligands for homogeneous catalysis which coordinate to late transition metals (Baumgartner & Marschner, 2014; Lee & Sekiguchi, 2010). Recently we described the synthesis and structures of some Pd, Mo and W complexes bearing Ge(II) and Sn(II) containing ligands (Kireenko *et al.*, 2012, 2013; Huang *et al.*, 2012, 2013).

The structure of the mixed-valence µ-oxo Sn_12_ title compound is shown in Fig. 1. The molecule comprises six distinct tin atom species with four different coordination numbers, namely 3, 4, 5, and 6. The three-coordinated Sn4, Sn5, Sn6 tin(II) atoms adopt a distorted trigonal pyramidal coordination geometry, with Sn—O bond lengths ranging from 2.064 (3) to 2.240 (3) Å and O—Sn—O angles narrower than 93.2 (1) °. The four-coordinated tin(II) atom Sn3 exhibits a distorted square pyramidal geometry with Sn—O distances lying within 2.109 (3)–2.475 (3) Å and *cis* O—Sn—O angles varying within 70.3 (1)–76.3 (1) °. The coordination polyhedron about the six-coordinated Sn2 tin(II) atom is a distorted octahedron; the apical positions are occupied by the N2 and O8 atoms, the best equatorial plane is provided by the O7, O8^i^, O9, O21 atoms (maximum displacement: 0.232 (3) Å for O7; symmetry code: (i) 2 - *x*, 2 - *y*, -*z*), with the metal atom displaced by 0.1462 (3) Å toward N7. The terminal Sn1 tin(IV) atom shows a trigonal bipyramidal coordination geometry, with the N1 nitrogen atom and the O6 µ_4_-oxygen atom occupying the axial positions, and the equatorial positions engaged by the Mo atoms of a Mo(CO)_5_ group and by the O atoms of an alkoxy ligand (). The Sn—Mo bond length is 2.7879 (5) Å. Among five Mo—C bonds, the Mo—C1 bond lengths (opposite to the Sn1 atom) is the shortest (1.986 (5) Å). Both µ_4_-oxygen atoms O6 and O8 have a significantly distorted tetrahedral tin environment with Sn—O—Sn angles ranging from 94.9 (1) to 130.1 (1)°. Two intramolecular C—H···O hydrogen bonds (Table 1) involving methylene carbon atoms are present. In the crystal, pairs of weak intermolecular C—H···O hydrogen bonds (Table 1) link centrosymmetrically related complex molecules into dimers.

### S2. Experimental

The title compound was obtained in 10% yield by the reaction of equimolar mixture of [C_5_H_3_N(CH_2_CPh_2_O)_2_]_2_Sn and Mo(CO)_5_*THF (generated at room temperature in THF *in situ* under UV irradiation of Mo(CO)_6_ in THF) in toluene solution. The crystals suitable for X-Ray analysis were obtained after recrystallization from toluene at room temperature.

### S3. Refinement

All non-hydrogen atoms were refined with anisotropic thermal parameters except for the toluene solvent molecules. The C141–C147 toluene molecule is disordered over two orientations about an inversion centre. The C(*sp^2^*)– C(*sp^2^*) and C(*sp^2^*)– C(*sp^3^*) bond distances of the C121–C127 and C131–C137 toluene molecules were constrained to be 1.400 (5) and 1.480 (5) Å, respectively, and isotropic displacement parameters set equal and refined as free variables were applied. All hydrogen atoms were placed in calculated positions and refined using a riding model, with C—H = 0.95-0.99 Å, and with *U*_iso_(H) = 1.2 *U*_eq_(C) or 1.5 *U*_eq_(C) for methyl H atoms. A rotating model was applied to the methyl groups. Nine outliers were omitted in the last cycles of refinement.

### Crystal data

**Table:**

[Mo_2_Sn_12_(C_33_H_27_NO_2_)_4_O_10_(CO)_10_]·7C_7_H_8_	*Z* = 1
*M**_r_* = 4579.42	*F*(000) = 2246
Triclinic, *P*1	*D*_x_ = 1.727 Mg m^−^^3^
Hall symbol: -P 1	Mo *K*α radiation, λ = 0.71073 Å
*a* = 15.8218 (17) Å	Cell parameters from 9901 reflections
*b* = 15.9933 (17) Å	θ = 2.4–30.5°
*c* = 18.806 (2) Å	µ = 1.88 mm^−^^1^
α = 94.833 (2)°	*T* = 173 K
β = 95.014 (2)°	Prism, colourless
γ = 110.605 (2)°	0.25 × 0.10 × 0.08 mm
*V* = 4403.5 (8) Å^3^	

### Data collection

**Table:**

Bruker SMART APEX II diffractometer	15393 independent reflections
Radiation source: fine-focus sealed tube	12647 reflections with *I* > 2σ(*I*)
Graphite monochromator	*R*_int_ = 0.028
ω scans	θ_max_ = 25.1°, θ_min_ = 2.3°
Absorption correction: multi-scan (*SADABS*; Bruker, 2008)	*h* = −18→18
*T*_min_ = 0.651, *T*_max_ = 0.864	*k* = −19→19
32165 measured reflections	*l* = −22→22

### Refinement

**Table:**

Refinement on *F*^2^	Primary atom site location: structure-invariant direct methods
Least-squares matrix: full	Secondary atom site location: difference Fourier map
*R*[*F*^2^ > 2σ(*F*^2^)] = 0.033	Hydrogen site location: inferred from neighbouring sites
*wR*(*F*^2^) = 0.081	H-atom parameters constrained
*S* = 1.02	*w* = 1/[σ^2^(*F*_o_^2^) + (0.0327*P*)^2^ + 7.0359*P*] where *P* = (*F*_o_^2^ + 2*F*_c_^2^)/3
15393 reflections	(Δ/σ)_max_ = 0.002
939 parameters	Δρ_max_ = 1.05 e Å^−^^3^
13 restraints	Δρ_min_ = −0.86 e Å^−^^3^

### Special details

**Table:**

Geometry. All e.s.d.'s (except the e.s.d. in the dihedral angle between two l.s. planes) are estimated using the full covariance matrix. The cell e.s.d.'s are taken into account individually in the estimation of e.s.d.'s in distances, angles and torsion angles; correlations between e.s.d.'s in cell parameters are only used when they are defined by crystal symmetry. An approximate (isotropic) treatment of cell e.s.d.'s is used for estimating e.s.d.'s involving l.s. planes.
Refinement. Refinement of *F*^2^ against ALL reflections. The weighted *R*-factor *wR* and goodness of fit *S* are based on *F*^2^, conventional *R*-factors *R* are based on *F*, with *F* set to zero for negative *F*^2^. The threshold expression of *F*^2^ > σ(*F*^2^) is used only for calculating *R*-factors(gt) *etc*. and is not relevant to the choice of reflections for refinement. *R*-factors based on *F*^2^ are statistically about twice as large as those based on *F*, and *R*- factors based on ALL data will be even larger.

### Fractional atomic coordinates and isotropic or equivalent isotropic displacement parameters (Å^2^)

**Table:**

	*x*	*y*	*z*	*U*_iso_*/*U*_eq_	Occ. (<1)
Sn1	0.685246 (19)	0.791596 (19)	0.170459 (15)	0.01787 (7)	
Sn2	0.966134 (19)	0.911141 (18)	−0.060628 (15)	0.01612 (7)	
Sn3	0.810971 (19)	0.952093 (19)	0.039582 (15)	0.01870 (7)	
Sn4	0.89930 (2)	1.117309 (19)	−0.097902 (16)	0.01963 (7)	
Sn5	0.92982 (2)	0.92791 (2)	0.192584 (15)	0.02104 (8)	
Sn6	0.85140 (2)	0.757667 (19)	0.060876 (16)	0.02163 (8)	
Mo1	0.53944 (3)	0.70788 (3)	0.06271 (2)	0.02364 (10)	
C1	0.4366 (3)	0.6569 (3)	−0.0160 (3)	0.0361 (12)	
O1	0.3815 (3)	0.6304 (3)	−0.0648 (2)	0.0605 (12)	
C2	0.5399 (3)	0.8310 (3)	0.0395 (2)	0.0317 (11)	
O2	0.5359 (3)	0.8959 (3)	0.0220 (2)	0.0490 (10)	
C3	0.6197 (3)	0.7062 (3)	−0.0167 (3)	0.0362 (12)	
O3	0.6577 (3)	0.7023 (3)	−0.0649 (2)	0.0582 (12)	
C4	0.4556 (3)	0.7000 (3)	0.1403 (3)	0.0328 (11)	
O4	0.4091 (2)	0.6921 (3)	0.1841 (2)	0.0467 (10)	
C5	0.5335 (4)	0.5822 (4)	0.0802 (3)	0.0416 (13)	
O5	0.5262 (3)	0.5108 (3)	0.0895 (2)	0.0677 (14)	
O6	0.80248 (19)	0.85657 (18)	0.11909 (15)	0.0203 (6)	
O7	0.85443 (18)	0.85519 (18)	−0.01250 (15)	0.0187 (6)	
O8	1.06470 (19)	0.97324 (18)	0.03098 (14)	0.0181 (6)	
O9	1.06823 (18)	0.98435 (18)	−0.11153 (14)	0.0176 (6)	
O10	1.03136 (19)	1.15737 (18)	−0.12387 (15)	0.0201 (6)	
N1	0.6241 (2)	0.7467 (2)	0.28026 (19)	0.0222 (8)	
N2	0.9094 (2)	0.8320 (2)	−0.17287 (18)	0.0201 (8)	
O11	0.78447 (19)	0.75398 (18)	0.21973 (16)	0.0228 (7)	
O12	0.7178 (2)	0.91510 (19)	0.22391 (17)	0.0280 (7)	
O21	0.98747 (19)	0.79539 (18)	−0.04078 (15)	0.0199 (6)	
O22	0.87601 (19)	1.01447 (19)	−0.18414 (15)	0.0229 (7)	
C11	0.7763 (3)	0.6773 (3)	0.2552 (2)	0.0225 (10)	
C12	0.6752 (3)	0.6218 (3)	0.2598 (2)	0.0250 (10)	
H12A	0.6411	0.6052	0.2108	0.030*	
H12B	0.6707	0.5655	0.2802	0.030*	
C13	0.6331 (3)	0.6737 (3)	0.3059 (2)	0.0265 (10)	
C14	0.6092 (3)	0.6517 (3)	0.3728 (3)	0.0351 (12)	
H14	0.6159	0.6002	0.3905	0.042*	
C15	0.5754 (4)	0.7059 (3)	0.4133 (3)	0.0410 (13)	
H15	0.5585	0.6917	0.4593	0.049*	
C16	0.5662 (3)	0.7810 (3)	0.3871 (3)	0.0362 (12)	
H16	0.5430	0.8186	0.4146	0.043*	
C17	0.5915 (3)	0.8002 (3)	0.3198 (2)	0.0251 (10)	
C18	0.5819 (3)	0.8784 (3)	0.2862 (3)	0.0263 (10)	
H18A	0.5509	0.9075	0.3180	0.032*	
H18B	0.5415	0.8549	0.2401	0.032*	
C19	0.6710 (3)	0.9520 (3)	0.2710 (2)	0.0254 (10)	
C21	1.0135 (3)	0.7384 (3)	−0.0902 (2)	0.0215 (9)	
C22	1.0447 (3)	0.7887 (3)	−0.1557 (2)	0.0212 (9)	
H22A	1.0872	0.8509	−0.1390	0.025*	
H22B	1.0769	0.7575	−0.1844	0.025*	
C23	0.9628 (3)	0.7907 (3)	−0.2014 (2)	0.0221 (10)	
C24	0.9369 (3)	0.7394 (3)	−0.2680 (2)	0.0309 (11)	
H24	0.9778	0.7160	−0.2889	0.037*	
C25	0.8516 (4)	0.7222 (3)	−0.3041 (3)	0.0405 (13)	
H25	0.8322	0.6858	−0.3494	0.049*	
C26	0.7956 (4)	0.7591 (3)	−0.2727 (3)	0.0341 (12)	
H26	0.7352	0.7448	−0.2951	0.041*	
C27	0.8256 (3)	0.8170 (3)	−0.2087 (2)	0.0237 (10)	
C28	0.7678 (3)	0.8649 (3)	−0.1809 (2)	0.0250 (10)	
H28A	0.7801	0.8762	−0.1278	0.030*	
H28B	0.7029	0.8264	−0.1941	0.030*	
C29	0.7865 (3)	0.9567 (3)	−0.2120 (2)	0.0232 (10)	
C31	0.8220 (3)	0.6238 (3)	0.2112 (2)	0.0241 (10)	
C32	0.7752 (3)	0.5447 (3)	0.1656 (2)	0.0295 (11)	
H32	0.7111	0.5169	0.1644	0.035*	
C33	0.8215 (4)	0.5056 (3)	0.1215 (3)	0.0349 (12)	
H33	0.7889	0.4511	0.0909	0.042*	
C34	0.9131 (3)	0.5455 (3)	0.1222 (3)	0.0321 (11)	
H34	0.9442	0.5194	0.0916	0.039*	
C35	0.9610 (3)	0.6241 (3)	0.1675 (3)	0.0316 (11)	
H35	1.0251	0.6513	0.1685	0.038*	
C36	0.9158 (3)	0.6626 (3)	0.2110 (2)	0.0265 (10)	
H36	0.9493	0.7168	0.2416	0.032*	
C41	0.8248 (3)	0.7055 (3)	0.3317 (2)	0.0264 (10)	
C42	0.8368 (4)	0.6409 (4)	0.3725 (3)	0.0388 (13)	
H42	0.8185	0.5800	0.3516	0.047*	
C43	0.8758 (4)	0.6661 (4)	0.4443 (3)	0.0536 (16)	
H43	0.8831	0.6219	0.4724	0.064*	
C44	0.9034 (4)	0.7534 (5)	0.4743 (3)	0.0563 (17)	
H44	0.9293	0.7696	0.5232	0.068*	
C45	0.8940 (4)	0.8174 (4)	0.4345 (3)	0.0454 (14)	
H45	0.9149	0.8784	0.4556	0.054*	
C46	0.8541 (3)	0.7944 (3)	0.3634 (3)	0.0330 (12)	
H46	0.8468	0.8394	0.3364	0.040*	
C51	0.6417 (3)	1.0224 (3)	0.2355 (2)	0.0256 (10)	
C52	0.5979 (3)	1.0710 (3)	0.2717 (3)	0.0334 (12)	
H52	0.5888	1.0632	0.3204	0.040*	
C53	0.5672 (4)	1.1308 (3)	0.2379 (3)	0.0425 (14)	
H53	0.5364	1.1628	0.2633	0.051*	
C54	0.5811 (4)	1.1440 (4)	0.1688 (3)	0.0441 (14)	
H54	0.5611	1.1859	0.1463	0.053*	
C55	0.6239 (4)	1.0965 (4)	0.1315 (3)	0.0425 (14)	
H55	0.6323	1.1048	0.0829	0.051*	
C56	0.6551 (3)	1.0360 (3)	0.1649 (3)	0.0353 (12)	
H56	0.6856	1.0041	0.1390	0.042*	
C61	0.7361 (3)	0.9971 (3)	0.3403 (3)	0.0275 (11)	
C62	0.8294 (3)	1.0312 (3)	0.3361 (3)	0.0423 (14)	
H62	0.8510	1.0251	0.2911	0.051*	
C63	0.8908 (4)	1.0735 (4)	0.3961 (3)	0.0516 (16)	
H63	0.9541	1.0957	0.3921	0.062*	
C64	0.8614 (4)	1.0843 (4)	0.4621 (3)	0.0487 (15)	
H64	0.9037	1.1133	0.5037	0.058*	
C65	0.7697 (4)	1.0521 (4)	0.4663 (3)	0.0448 (14)	
H65	0.7483	1.0595	0.5111	0.054*	
C66	0.7083 (3)	1.0092 (3)	0.4066 (3)	0.0350 (12)	
H66	0.6451	0.9875	0.4111	0.042*	
C71	1.0894 (3)	0.7166 (3)	−0.0489 (2)	0.0236 (10)	
C72	1.1810 (3)	0.7605 (3)	−0.0534 (3)	0.0314 (11)	
H72	1.1988	0.8036	−0.0863	0.038*	
C73	1.2470 (3)	0.7426 (4)	−0.0110 (3)	0.0420 (13)	
H73	1.3096	0.7737	−0.0144	0.050*	
C74	1.2218 (4)	0.6795 (4)	0.0364 (3)	0.0386 (13)	
H74	1.2670	0.6666	0.0652	0.046*	
C75	1.1315 (3)	0.6355 (3)	0.0416 (3)	0.0345 (12)	
H75	1.1141	0.5922	0.0744	0.041*	
C76	1.0654 (3)	0.6536 (3)	−0.0005 (2)	0.0296 (11)	
H76	1.0029	0.6227	0.0036	0.036*	
C81	0.9330 (3)	0.6513 (3)	−0.1195 (2)	0.0213 (9)	
C82	0.8440 (3)	0.6405 (3)	−0.1121 (2)	0.0269 (10)	
H82	0.8309	0.6863	−0.0849	0.032*	
C83	0.7733 (3)	0.5633 (3)	−0.1441 (3)	0.0346 (12)	
H83	0.7123	0.5564	−0.1383	0.042*	
C84	0.7909 (4)	0.4966 (3)	−0.1842 (3)	0.0398 (13)	
H84	0.7424	0.4435	−0.2056	0.048*	
C85	0.8793 (3)	0.5079 (3)	−0.1928 (3)	0.0334 (12)	
H85	0.8917	0.4627	−0.2214	0.040*	
C86	0.9505 (3)	0.5837 (3)	−0.1606 (2)	0.0273 (10)	
H86	1.0115	0.5900	−0.1663	0.033*	
C91	0.7179 (3)	0.9999 (3)	−0.1916 (2)	0.0238 (10)	
C92	0.7089 (3)	1.0682 (3)	−0.2292 (3)	0.0344 (12)	
H92	0.7404	1.0825	−0.2699	0.041*	
C93	0.6554 (4)	1.1154 (4)	−0.2085 (3)	0.0409 (13)	
H93	0.6500	1.1617	−0.2350	0.049*	
C94	0.6096 (3)	1.0957 (4)	−0.1494 (3)	0.0391 (13)	
H94	0.5734	1.1288	−0.1347	0.047*	
C95	0.6164 (3)	1.0282 (3)	−0.1121 (3)	0.0359 (12)	
H95	0.5850	1.0145	−0.0713	0.043*	
C96	0.6694 (3)	0.9795 (3)	−0.1336 (2)	0.0284 (11)	
H96	0.6722	0.9315	−0.1081	0.034*	
C101	0.7761 (3)	0.9359 (3)	−0.2946 (2)	0.0252 (10)	
C102	0.6914 (3)	0.8866 (3)	−0.3321 (3)	0.0345 (12)	
H102	0.6390	0.8720	−0.3075	0.041*	
C103	0.6819 (4)	0.8583 (4)	−0.4049 (3)	0.0422 (13)	
H103	0.6234	0.8241	−0.4299	0.051*	
C104	0.7567 (4)	0.8795 (4)	−0.4409 (3)	0.0483 (15)	
H104	0.7503	0.8589	−0.4907	0.058*	
C105	0.8416 (4)	0.9311 (4)	−0.4048 (3)	0.0456 (14)	
H105	0.8934	0.9473	−0.4300	0.055*	
C106	0.8510 (3)	0.9592 (3)	−0.3317 (3)	0.0325 (11)	
H106	0.9094	0.9947	−0.3070	0.039*	
C111	0.0557 (5)	0.5705 (5)	0.6447 (4)	0.0699 (19)*	
C112	0.0782 (5)	0.6529 (5)	0.6126 (4)	0.071 (2)*	
H112	0.0447	0.6568	0.5694	0.085*	
C113	0.1486 (5)	0.7240 (5)	0.6465 (4)	0.0655 (18)*	
H113	0.1663	0.7781	0.6248	0.079*	
C114	0.1964 (5)	0.7230 (5)	0.7109 (4)	0.077 (2)*	
H114	0.2440	0.7759	0.7338	0.092*	
C115	0.1749 (5)	0.6470 (5)	0.7404 (4)	0.075 (2)*	
H115	0.2078	0.6452	0.7847	0.090*	
C116	0.1042 (4)	0.5694 (4)	0.7067 (3)	0.0581 (17)*	
H116	0.0903	0.5151	0.7280	0.070*	
C117	−0.0207 (7)	0.4918 (7)	0.6110 (5)	0.124 (4)*	
H11C	−0.0617	0.4679	0.6467	0.186*	
H11B	0.0017	0.4456	0.5920	0.186*	
H11A	−0.0538	0.5086	0.5716	0.186*	
C121	0.1876 (6)	0.7334 (7)	0.3756 (5)	0.1144 (13)*	
C122	0.1926 (6)	0.7164 (6)	0.4470 (5)	0.1144 (13)*	
H122	0.1552	0.7329	0.4780	0.137*	
C123	0.2512 (6)	0.6758 (6)	0.4737 (5)	0.1144 (13)*	
H123	0.2535	0.6653	0.5226	0.137*	
C124	0.3073 (7)	0.6499 (6)	0.4294 (5)	0.1144 (13)*	
H124	0.3483	0.6227	0.4467	0.137*	
C125	0.2983 (7)	0.6672 (6)	0.3586 (5)	0.1144 (13)*	
H125	0.3354	0.6509	0.3274	0.137*	
C126	0.2399 (6)	0.7061 (6)	0.3296 (5)	0.1144 (13)*	
H126	0.2356	0.7140	0.2801	0.137*	
C127	0.1240 (6)	0.7751 (6)	0.3491 (5)	0.1144 (13)*	
H12C	0.1172	0.7685	0.2964	0.172*	
H12D	0.1476	0.8392	0.3678	0.172*	
H12E	0.0647	0.7456	0.3654	0.172*	
C131	0.4604 (7)	0.4059 (7)	0.3531 (5)	0.1180 (13)*	
C132	0.4439 (7)	0.4062 (7)	0.2790 (5)	0.1180 (13)*	
H132	0.3966	0.4237	0.2586	0.142*	
C133	0.4999 (6)	0.3798 (7)	0.2365 (5)	0.1180 (13)*	
H133	0.4852	0.3717	0.1858	0.142*	
C134	0.5760 (6)	0.3647 (7)	0.2649 (5)	0.1180 (13)*	
H134	0.6151	0.3504	0.2345	0.142*	
C135	0.5938 (7)	0.3710 (7)	0.3392 (5)	0.1180 (13)*	
H135	0.6462	0.3616	0.3599	0.142*	
C136	0.5357 (6)	0.3908 (7)	0.3837 (5)	0.1180 (13)*	
H136	0.5477	0.3939	0.4345	0.142*	
C137	0.4025 (7)	0.4285 (7)	0.4018 (5)	0.1180 (13)*	
H13A	0.4404	0.4667	0.4448	0.177*	
H13B	0.3597	0.3732	0.4159	0.177*	
H13C	0.3685	0.4609	0.3775	0.177*	
C141	0.5280 (4)	0.0816 (4)	0.4727 (3)	0.0522 (15)*	
H141	0.5474	0.1377	0.4539	0.063*	0.50
C142	0.5665 (4)	0.0736 (4)	0.5390 (3)	0.0549 (16)*	
H142	0.6124	0.1247	0.5662	0.066*	
C143	0.5395 (4)	−0.0071 (4)	0.5663 (3)	0.0556 (16)*	
H143	0.5671	−0.0120	0.6119	0.067*	
C147	0.4373 (9)	−0.1710 (9)	0.5552 (7)	0.064 (4)*	0.50
H14A	0.4419	−0.2176	0.5201	0.096*	0.50
H14B	0.3735	−0.1854	0.5630	0.096*	0.50
H14C	0.4737	−0.1684	0.6008	0.096*	0.50

### Atomic displacement parameters (Å^2^)

**Table:**

	*U*^11^	*U*^22^	*U*^33^	*U*^12^	*U*^13^	*U*^23^
Sn1	0.01573 (15)	0.01795 (15)	0.01960 (15)	0.00602 (12)	0.00312 (12)	−0.00007 (11)
Sn2	0.01583 (15)	0.01599 (15)	0.01729 (15)	0.00685 (12)	0.00359 (11)	−0.00015 (11)
Sn3	0.01641 (15)	0.02035 (16)	0.02130 (16)	0.00919 (12)	0.00340 (12)	0.00085 (12)
Sn4	0.01893 (16)	0.02015 (16)	0.02271 (16)	0.01062 (13)	0.00291 (12)	0.00232 (12)
Sn5	0.02239 (16)	0.02407 (16)	0.01741 (15)	0.00931 (13)	0.00342 (12)	0.00168 (12)
Sn6	0.02153 (16)	0.01691 (15)	0.02576 (17)	0.00640 (13)	0.00212 (13)	0.00224 (12)
Mo1	0.0186 (2)	0.0253 (2)	0.0252 (2)	0.00811 (17)	−0.00110 (16)	−0.00314 (16)
C1	0.023 (3)	0.043 (3)	0.041 (3)	0.015 (2)	−0.003 (2)	−0.009 (2)
O1	0.040 (2)	0.078 (3)	0.056 (3)	0.026 (2)	−0.021 (2)	−0.025 (2)
C2	0.033 (3)	0.039 (3)	0.022 (3)	0.014 (2)	0.001 (2)	−0.001 (2)
O2	0.062 (3)	0.043 (2)	0.050 (2)	0.027 (2)	0.004 (2)	0.0147 (19)
C3	0.022 (3)	0.044 (3)	0.036 (3)	0.008 (2)	−0.005 (2)	−0.005 (2)
O3	0.037 (2)	0.091 (3)	0.040 (2)	0.018 (2)	0.0116 (19)	−0.009 (2)
C4	0.023 (3)	0.033 (3)	0.038 (3)	0.006 (2)	0.000 (2)	0.001 (2)
O4	0.033 (2)	0.055 (2)	0.046 (2)	0.0068 (18)	0.0154 (19)	0.0027 (19)
C5	0.048 (3)	0.035 (3)	0.034 (3)	0.011 (3)	−0.009 (3)	−0.008 (2)
O5	0.106 (4)	0.033 (2)	0.060 (3)	0.029 (2)	−0.019 (3)	−0.006 (2)
O6	0.0181 (15)	0.0214 (16)	0.0218 (16)	0.0060 (13)	0.0087 (12)	0.0031 (12)
O7	0.0181 (15)	0.0187 (15)	0.0211 (15)	0.0082 (12)	0.0064 (12)	0.0014 (12)
O8	0.0194 (15)	0.0173 (15)	0.0184 (15)	0.0080 (12)	0.0019 (12)	0.0009 (12)
O9	0.0177 (15)	0.0169 (15)	0.0178 (15)	0.0051 (12)	0.0055 (12)	0.0020 (12)
O10	0.0194 (16)	0.0187 (15)	0.0210 (16)	0.0059 (13)	0.0029 (12)	−0.0001 (12)
N1	0.0158 (19)	0.024 (2)	0.025 (2)	0.0040 (16)	0.0032 (15)	0.0012 (16)
N2	0.0210 (19)	0.0175 (18)	0.0220 (19)	0.0076 (16)	0.0030 (16)	0.0003 (15)
O11	0.0173 (15)	0.0195 (16)	0.0326 (18)	0.0072 (13)	0.0029 (13)	0.0069 (13)
O12	0.0273 (18)	0.0206 (16)	0.0364 (19)	0.0085 (14)	0.0134 (15)	−0.0036 (14)
O21	0.0242 (16)	0.0193 (15)	0.0204 (15)	0.0131 (13)	0.0037 (13)	0.0008 (12)
O22	0.0189 (16)	0.0283 (17)	0.0227 (16)	0.0114 (13)	0.0021 (13)	−0.0027 (13)
C11	0.023 (2)	0.019 (2)	0.024 (2)	0.0067 (19)	0.0014 (19)	0.0026 (18)
C12	0.028 (3)	0.021 (2)	0.027 (2)	0.009 (2)	0.003 (2)	0.0020 (19)
C13	0.023 (2)	0.026 (3)	0.029 (3)	0.007 (2)	0.003 (2)	0.004 (2)
C14	0.038 (3)	0.034 (3)	0.035 (3)	0.011 (2)	0.014 (2)	0.012 (2)
C15	0.050 (3)	0.042 (3)	0.033 (3)	0.014 (3)	0.021 (3)	0.012 (2)
C16	0.040 (3)	0.034 (3)	0.034 (3)	0.010 (2)	0.021 (2)	0.002 (2)
C17	0.017 (2)	0.026 (2)	0.030 (3)	0.0048 (19)	0.0077 (19)	0.002 (2)
C18	0.021 (2)	0.031 (3)	0.030 (3)	0.013 (2)	0.007 (2)	−0.001 (2)
C19	0.023 (2)	0.022 (2)	0.032 (3)	0.009 (2)	0.007 (2)	−0.0019 (19)
C21	0.022 (2)	0.019 (2)	0.026 (2)	0.0110 (19)	0.0062 (19)	0.0021 (18)
C22	0.020 (2)	0.020 (2)	0.025 (2)	0.0099 (19)	0.0044 (19)	−0.0012 (18)
C23	0.025 (2)	0.022 (2)	0.023 (2)	0.0102 (19)	0.0077 (19)	0.0064 (18)
C24	0.038 (3)	0.032 (3)	0.027 (3)	0.018 (2)	0.007 (2)	−0.001 (2)
C25	0.055 (4)	0.043 (3)	0.027 (3)	0.028 (3)	−0.008 (3)	−0.009 (2)
C26	0.039 (3)	0.030 (3)	0.032 (3)	0.017 (2)	−0.012 (2)	−0.007 (2)
C27	0.026 (2)	0.022 (2)	0.024 (2)	0.010 (2)	−0.0004 (19)	0.0044 (19)
C28	0.024 (2)	0.027 (2)	0.024 (2)	0.012 (2)	−0.0030 (19)	0.0007 (19)
C29	0.023 (2)	0.025 (2)	0.022 (2)	0.010 (2)	−0.0006 (19)	0.0018 (18)
C31	0.028 (3)	0.023 (2)	0.025 (2)	0.014 (2)	0.003 (2)	0.0044 (19)
C32	0.026 (3)	0.032 (3)	0.032 (3)	0.013 (2)	0.003 (2)	0.000 (2)
C33	0.041 (3)	0.027 (3)	0.036 (3)	0.013 (2)	0.005 (2)	0.000 (2)
C34	0.043 (3)	0.028 (3)	0.034 (3)	0.023 (2)	0.013 (2)	0.005 (2)
C35	0.028 (3)	0.031 (3)	0.041 (3)	0.015 (2)	0.008 (2)	0.008 (2)
C36	0.028 (3)	0.024 (2)	0.028 (3)	0.010 (2)	0.006 (2)	0.0014 (19)
C41	0.022 (2)	0.033 (3)	0.024 (2)	0.011 (2)	0.0034 (19)	−0.002 (2)
C42	0.047 (3)	0.041 (3)	0.031 (3)	0.022 (3)	−0.002 (2)	0.002 (2)
C43	0.059 (4)	0.068 (4)	0.036 (3)	0.027 (3)	−0.004 (3)	0.013 (3)
C44	0.050 (4)	0.077 (5)	0.025 (3)	0.008 (3)	−0.004 (3)	−0.006 (3)
C45	0.037 (3)	0.050 (4)	0.038 (3)	0.004 (3)	0.010 (3)	−0.011 (3)
C46	0.029 (3)	0.036 (3)	0.030 (3)	0.007 (2)	0.008 (2)	−0.003 (2)
C51	0.021 (2)	0.022 (2)	0.032 (3)	0.0054 (19)	0.005 (2)	0.0018 (19)
C52	0.037 (3)	0.031 (3)	0.037 (3)	0.016 (2)	0.012 (2)	0.003 (2)
C53	0.044 (3)	0.034 (3)	0.060 (4)	0.024 (3)	0.016 (3)	0.005 (3)
C54	0.041 (3)	0.036 (3)	0.058 (4)	0.015 (3)	0.003 (3)	0.018 (3)
C55	0.042 (3)	0.042 (3)	0.039 (3)	0.007 (3)	0.011 (3)	0.012 (3)
C56	0.034 (3)	0.031 (3)	0.040 (3)	0.009 (2)	0.010 (2)	0.003 (2)
C61	0.024 (2)	0.019 (2)	0.040 (3)	0.010 (2)	0.005 (2)	0.000 (2)
C62	0.027 (3)	0.040 (3)	0.054 (4)	0.010 (2)	0.009 (3)	−0.018 (3)
C63	0.022 (3)	0.048 (4)	0.075 (4)	0.012 (3)	−0.009 (3)	−0.021 (3)
C64	0.044 (4)	0.045 (3)	0.048 (4)	0.012 (3)	−0.012 (3)	−0.011 (3)
C65	0.048 (4)	0.047 (3)	0.033 (3)	0.012 (3)	−0.001 (3)	0.000 (2)
C66	0.031 (3)	0.037 (3)	0.034 (3)	0.009 (2)	0.006 (2)	−0.002 (2)
C71	0.029 (3)	0.019 (2)	0.026 (2)	0.014 (2)	0.001 (2)	−0.0062 (18)
C72	0.029 (3)	0.027 (3)	0.041 (3)	0.013 (2)	0.003 (2)	0.005 (2)
C73	0.023 (3)	0.046 (3)	0.059 (4)	0.014 (2)	0.002 (3)	0.008 (3)
C74	0.036 (3)	0.045 (3)	0.040 (3)	0.023 (3)	−0.003 (2)	0.002 (2)
C75	0.040 (3)	0.039 (3)	0.031 (3)	0.021 (3)	0.005 (2)	0.009 (2)
C76	0.030 (3)	0.033 (3)	0.030 (3)	0.016 (2)	0.004 (2)	0.003 (2)
C81	0.024 (2)	0.023 (2)	0.020 (2)	0.0107 (19)	0.0042 (19)	0.0060 (18)
C82	0.026 (3)	0.026 (2)	0.028 (3)	0.009 (2)	0.005 (2)	0.000 (2)
C83	0.025 (3)	0.035 (3)	0.039 (3)	0.005 (2)	0.006 (2)	−0.001 (2)
C84	0.035 (3)	0.028 (3)	0.043 (3)	−0.001 (2)	0.002 (2)	−0.005 (2)
C85	0.037 (3)	0.025 (3)	0.036 (3)	0.011 (2)	0.002 (2)	−0.002 (2)
C86	0.029 (3)	0.025 (2)	0.028 (3)	0.010 (2)	0.003 (2)	0.003 (2)
C91	0.015 (2)	0.028 (2)	0.026 (2)	0.0090 (19)	−0.0061 (19)	−0.0020 (19)
C92	0.038 (3)	0.039 (3)	0.033 (3)	0.022 (2)	0.009 (2)	0.008 (2)
C93	0.046 (3)	0.044 (3)	0.047 (3)	0.033 (3)	0.008 (3)	0.016 (3)
C94	0.034 (3)	0.046 (3)	0.048 (3)	0.027 (3)	0.011 (3)	0.003 (3)
C95	0.030 (3)	0.043 (3)	0.036 (3)	0.014 (2)	0.008 (2)	0.004 (2)
C96	0.024 (2)	0.032 (3)	0.031 (3)	0.012 (2)	0.002 (2)	0.004 (2)
C101	0.030 (3)	0.030 (3)	0.019 (2)	0.017 (2)	−0.003 (2)	0.0015 (19)
C102	0.031 (3)	0.041 (3)	0.032 (3)	0.017 (2)	−0.005 (2)	−0.003 (2)
C103	0.037 (3)	0.054 (4)	0.033 (3)	0.021 (3)	−0.013 (2)	−0.009 (3)
C104	0.059 (4)	0.068 (4)	0.020 (3)	0.030 (3)	−0.003 (3)	−0.005 (3)
C105	0.044 (3)	0.065 (4)	0.026 (3)	0.018 (3)	0.006 (2)	0.001 (3)
C106	0.033 (3)	0.035 (3)	0.028 (3)	0.011 (2)	0.001 (2)	−0.001 (2)

### Geometric parameters (Å, º)

**Table:**

Sn1—O12	2.009 (3)	C54—C55	1.371 (8)
Sn1—O11	2.047 (3)	C54—H54	0.9500
Sn1—O6	2.140 (3)	C55—C56	1.400 (7)
Sn1—N1	2.414 (3)	C55—H55	0.9500
Sn1—Mo1	2.7879 (5)	C56—H56	0.9500
Sn2—O9	2.006 (3)	C61—C66	1.379 (7)
Sn2—O7	2.012 (3)	C61—C62	1.395 (7)
Sn2—O21	2.053 (3)	C62—C63	1.375 (7)
Sn2—O8^i^	2.116 (3)	C62—H62	0.9500
Sn2—O8	2.130 (3)	C63—C64	1.382 (8)
Sn2—N2	2.298 (3)	C63—H63	0.9500
Sn2—Sn2^i^	3.2809 (6)	C64—C65	1.369 (8)
Sn3—O7	2.109 (3)	C64—H64	0.9500
Sn3—O9^i^	2.113 (3)	C65—C66	1.373 (7)
Sn3—O6	2.205 (3)	C65—H65	0.9500
Sn3—O8^i^	2.475 (3)	C66—H66	0.9500
Sn4—O10	2.075 (3)	C71—C72	1.384 (6)
Sn4—O22	2.119 (3)	C71—C76	1.390 (6)
Sn4—O8^i^	2.184 (3)	C72—C73	1.383 (7)
Sn5—O10^i^	2.083 (3)	C72—H72	0.9500
Sn5—O9^i^	2.152 (3)	C73—C74	1.378 (7)
Sn5—O6	2.214 (3)	C73—H73	0.9500
Sn6—O10^i^	2.064 (3)	C74—C75	1.370 (7)
Sn6—O7	2.159 (3)	C74—H74	0.9500
Sn6—O6	2.240 (3)	C75—C76	1.383 (7)
Mo1—C1	1.986 (5)	C75—H75	0.9500
Mo1—C5	2.035 (6)	C76—H76	0.9500
Mo1—C4	2.040 (5)	C81—C82	1.378 (6)
Mo1—C3	2.047 (5)	C81—C86	1.397 (6)
Mo1—C2	2.050 (5)	C82—C83	1.386 (6)
C1—O1	1.147 (6)	C82—H82	0.9500
C2—O2	1.136 (6)	C83—C84	1.376 (7)
C3—O3	1.141 (6)	C83—H83	0.9500
C4—O4	1.137 (6)	C84—C85	1.373 (7)
C5—O5	1.136 (6)	C84—H84	0.9500
O8—Sn2^i^	2.116 (3)	C85—C86	1.380 (6)
O8—Sn4^i^	2.184 (3)	C85—H85	0.9500
O8—Sn3^i^	2.475 (3)	C86—H86	0.9500
O9—Sn3^i^	2.113 (3)	C91—C96	1.385 (6)
O9—Sn5^i^	2.152 (3)	C91—C92	1.389 (6)
O10—Sn6^i^	2.064 (3)	C92—C93	1.378 (7)
O10—Sn5^i^	2.083 (3)	C92—H92	0.9500
N1—C13	1.347 (6)	C93—C94	1.378 (7)
N1—C17	1.351 (6)	C93—H93	0.9500
N2—C23	1.361 (5)	C94—C95	1.365 (7)
N2—C27	1.364 (5)	C94—H94	0.9500
O11—C11	1.416 (5)	C95—C96	1.392 (7)
O12—C19	1.420 (5)	C95—H95	0.9500
O21—C21	1.433 (5)	C96—H96	0.9500
O22—C29	1.415 (5)	C101—C106	1.382 (7)
C11—C41	1.522 (6)	C101—C102	1.383 (6)
C11—C31	1.536 (6)	C102—C103	1.383 (7)
C11—C12	1.547 (6)	C102—H102	0.9500
C12—C13	1.501 (6)	C103—C104	1.368 (8)
C12—H12A	0.9900	C103—H103	0.9500
C12—H12B	0.9900	C104—C105	1.383 (8)
C13—C14	1.383 (6)	C104—H104	0.9500
C14—C15	1.380 (7)	C105—C106	1.388 (7)
C14—H14	0.9500	C105—H105	0.9500
C15—C16	1.384 (7)	C106—H106	0.9500
C15—H15	0.9500	C111—C116	1.344 (9)
C16—C17	1.386 (6)	C111—C112	1.438 (9)
C16—H16	0.9500	C111—C117	1.453 (11)
C17—C18	1.496 (6)	C112—C113	1.343 (9)
C18—C19	1.553 (6)	C112—H112	0.9500
C18—H18A	0.9900	C113—C114	1.374 (9)
C18—H18B	0.9900	C113—H113	0.9500
C19—C61	1.537 (6)	C114—C115	1.326 (10)
C19—C51	1.539 (6)	C114—H114	0.9500
C21—C71	1.532 (6)	C115—C116	1.400 (9)
C21—C81	1.538 (6)	C115—H115	0.9500
C21—C22	1.549 (6)	C116—H116	0.9500
C22—C23	1.503 (6)	C117—H11C	0.9800
C22—H22A	0.9900	C117—H11B	0.9800
C22—H22B	0.9900	C117—H11A	0.9800
C23—C24	1.383 (6)	C121—C122	1.392 (5)
C24—C25	1.381 (7)	C121—C126	1.393 (5)
C24—H24	0.9500	C121—C127	1.466 (5)
C25—C26	1.371 (7)	C122—C123	1.391 (5)
C25—H25	0.9500	C122—H122	0.9500
C26—C27	1.392 (6)	C123—C124	1.407 (5)
C26—H26	0.9500	C123—H123	0.9500
C27—C28	1.486 (6)	C124—C125	1.388 (5)
C28—C29	1.567 (6)	C124—H124	0.9500
C28—H28A	0.9900	C125—C126	1.383 (5)
C28—H28B	0.9900	C125—H125	0.9500
C29—C91	1.536 (6)	C126—H126	0.9500
C29—C101	1.542 (6)	C127—H12C	0.9800
C31—C32	1.390 (6)	C127—H12D	0.9800
C31—C36	1.394 (6)	C127—H12E	0.9800
C32—C33	1.399 (7)	C131—C136	1.384 (5)
C32—H32	0.9500	C131—C132	1.395 (5)
C33—C34	1.361 (7)	C131—C137	1.462 (12)
C33—H33	0.9500	C132—C133	1.390 (5)
C34—C35	1.384 (7)	C132—H132	0.9500
C34—H34	0.9500	C133—C134	1.380 (5)
C35—C36	1.373 (6)	C133—H133	0.9500
C35—H35	0.9500	C134—C135	1.388 (5)
C36—H36	0.9500	C134—H134	0.9500
C41—C46	1.391 (6)	C135—C136	1.393 (5)
C41—C42	1.392 (7)	C135—H135	0.9500
C42—C43	1.399 (7)	C136—H136	0.9500
C42—H42	0.9500	C137—H13A	0.9800
C43—C44	1.361 (8)	C137—H13B	0.9800
C43—H43	0.9500	C137—H13C	0.9800
C44—C45	1.358 (8)	C141—C142	1.375 (8)
C44—H44	0.9500	C141—C143^ii^	1.386 (8)
C45—C46	1.388 (7)	C141—H141	0.9500
C45—H45	0.9500	C142—C143	1.369 (8)
C46—H46	0.9500	C142—H142	0.9500
C51—C56	1.385 (6)	C143—C141^ii^	1.386 (8)
C51—C52	1.386 (6)	C143—H143	0.9500
C52—C53	1.391 (7)	C147—C141^ii^	1.498 (13)
C52—H52	0.9500	C147—H14A	0.9800
C53—C54	1.359 (8)	C147—H14B	0.9800
C53—H53	0.9500	C147—H14C	0.9800
			
O12—Sn1—O11	99.97 (12)	C45—C44—H44	119.8
O12—Sn1—O6	83.14 (11)	C43—C44—H44	119.8
O11—Sn1—O6	75.96 (11)	C44—C45—C46	120.5 (5)
O12—Sn1—N1	82.07 (12)	C44—C45—H45	119.8
O11—Sn1—N1	78.62 (12)	C46—C45—H45	119.8
O6—Sn1—N1	147.76 (11)	C45—C46—C41	120.3 (5)
O12—Sn1—Mo1	125.85 (9)	C45—C46—H46	119.8
O11—Sn1—Mo1	134.16 (8)	C41—C46—H46	119.8
O6—Sn1—Mo1	107.49 (8)	C56—C51—C52	118.2 (4)
N1—Sn1—Mo1	104.41 (8)	C56—C51—C19	120.5 (4)
O9—Sn2—O7	169.60 (11)	C52—C51—C19	121.3 (4)
O9—Sn2—O21	108.06 (11)	C51—C52—C53	120.9 (5)
O7—Sn2—O21	82.30 (11)	C51—C52—H52	119.6
O9—Sn2—O8^i^	90.44 (11)	C53—C52—H52	119.6
O7—Sn2—O8^i^	80.06 (11)	C54—C53—C52	120.4 (5)
O21—Sn2—O8^i^	153.54 (11)	C54—C53—H53	119.8
O9—Sn2—O8	81.62 (11)	C52—C53—H53	119.8
O7—Sn2—O8	100.48 (11)	C53—C54—C55	119.9 (5)
O21—Sn2—O8	85.22 (11)	C53—C54—H54	120.1
O8^i^—Sn2—O8	78.81 (11)	C55—C54—H54	120.1
O9—Sn2—N2	83.69 (11)	C54—C55—C56	120.3 (5)
O7—Sn2—N2	97.19 (12)	C54—C55—H55	119.8
O21—Sn2—N2	82.47 (11)	C56—C55—H55	119.8
O8^i^—Sn2—N2	119.15 (11)	C51—C56—C55	120.3 (5)
O8—Sn2—N2	156.87 (11)	C51—C56—H56	119.9
O9—Sn2—Sn2^i^	84.85 (8)	C55—C56—H56	119.9
O7—Sn2—Sn2^i^	90.39 (8)	C66—C61—C62	117.2 (5)
O21—Sn2—Sn2^i^	121.55 (8)	C66—C61—C19	124.3 (4)
O8^i^—Sn2—Sn2^i^	39.57 (7)	C62—C61—C19	118.5 (4)
O8—Sn2—Sn2^i^	39.25 (7)	C63—C62—C61	121.1 (5)
N2—Sn2—Sn2^i^	155.68 (8)	C63—C62—H62	119.5
O7—Sn3—O9^i^	95.76 (11)	C61—C62—H62	119.5
O7—Sn3—O6	76.64 (10)	C62—C63—C64	120.8 (5)
O9^i^—Sn3—O6	76.33 (10)	C62—C63—H63	119.6
O7—Sn3—O8^i^	70.30 (10)	C64—C63—H63	119.6
O9^i^—Sn3—O8^i^	71.76 (10)	C65—C64—C63	118.3 (5)
O6—Sn3—O8^i^	130.61 (10)	C65—C64—H64	120.8
O10—Sn4—O22	82.66 (11)	C63—C64—H64	120.8
O10—Sn4—O8^i^	86.82 (11)	C64—C65—C66	121.1 (5)
O22—Sn4—O8^i^	85.41 (11)	C64—C65—H65	119.5
O10^i^—Sn5—O9^i^	91.93 (11)	C66—C65—H65	119.5
O10^i^—Sn5—O6	78.14 (11)	C65—C66—C61	121.6 (5)
O9^i^—Sn5—O6	75.37 (10)	C65—C66—H66	119.2
O10^i^—Sn6—O7	93.17 (11)	C61—C66—H66	119.2
O10^i^—Sn6—O6	77.92 (11)	C72—C71—C76	118.1 (4)
O7—Sn6—O6	74.91 (10)	C72—C71—C21	123.5 (4)
C1—Mo1—C5	90.9 (2)	C76—C71—C21	118.2 (4)
C1—Mo1—C4	93.3 (2)	C73—C72—C71	121.1 (5)
C5—Mo1—C4	87.0 (2)	C73—C72—H72	119.5
C1—Mo1—C3	84.9 (2)	C71—C72—H72	119.5
C5—Mo1—C3	89.5 (2)	C74—C73—C72	119.9 (5)
C4—Mo1—C3	176.0 (2)	C74—C73—H73	120.0
C1—Mo1—C2	85.6 (2)	C72—C73—H73	120.0
C5—Mo1—C2	176.5 (2)	C75—C74—C73	119.8 (5)
C4—Mo1—C2	93.7 (2)	C75—C74—H74	120.1
C3—Mo1—C2	89.7 (2)	C73—C74—H74	120.1
C1—Mo1—Sn1	175.92 (15)	C74—C75—C76	120.4 (5)
C5—Mo1—Sn1	93.13 (14)	C74—C75—H75	119.8
C4—Mo1—Sn1	87.44 (14)	C76—C75—H75	119.8
C3—Mo1—Sn1	94.62 (13)	C75—C76—C71	120.7 (5)
C2—Mo1—Sn1	90.32 (13)	C75—C76—H76	119.7
O1—C1—Mo1	175.1 (5)	C71—C76—H76	119.7
O2—C2—Mo1	174.7 (4)	C82—C81—C86	118.8 (4)
O3—C3—Mo1	174.1 (4)	C82—C81—C21	122.8 (4)
O4—C4—Mo1	177.1 (5)	C86—C81—C21	118.2 (4)
O5—C5—Mo1	177.1 (5)	C81—C82—C83	120.5 (4)
Sn1—O6—Sn3	125.55 (13)	C81—C82—H82	119.8
Sn1—O6—Sn5	115.29 (12)	C83—C82—H82	119.8
Sn3—O6—Sn5	101.54 (11)	C84—C83—C82	120.6 (5)
Sn1—O6—Sn6	112.17 (12)	C84—C83—H83	119.7
Sn3—O6—Sn6	101.22 (11)	C82—C83—H83	119.7
Sn5—O6—Sn6	96.48 (11)	C85—C84—C83	119.1 (5)
Sn2—O7—Sn3	112.51 (12)	C85—C84—H84	120.4
Sn2—O7—Sn6	120.20 (13)	C83—C84—H84	120.4
Sn3—O7—Sn6	107.23 (12)	C84—C85—C86	121.0 (5)
Sn2^i^—O8—Sn2	101.19 (11)	C84—C85—H85	119.5
Sn2^i^—O8—Sn4^i^	130.09 (13)	C86—C85—H85	119.5
Sn2—O8—Sn4^i^	116.31 (12)	C85—C86—C81	119.9 (4)
Sn2^i^—O8—Sn3^i^	96.23 (10)	C85—C86—H86	120.0
Sn2—O8—Sn3^i^	94.86 (10)	C81—C86—H86	120.0
Sn4^i^—O8—Sn3^i^	111.20 (11)	C96—C91—C92	117.7 (4)
Sn2—O9—Sn3^i^	111.21 (12)	C96—C91—C29	123.2 (4)
Sn2—O9—Sn5^i^	130.83 (14)	C92—C91—C29	118.9 (4)
Sn3^i^—O9—Sn5^i^	106.75 (11)	C93—C92—C91	121.3 (5)
Sn6^i^—O10—Sn4	127.47 (14)	C93—C92—H92	119.3
Sn6^i^—O10—Sn5^i^	106.46 (12)	C91—C92—H92	119.3
Sn4—O10—Sn5^i^	120.31 (13)	C94—C93—C92	120.1 (5)
C13—N1—C17	120.3 (4)	C94—C93—H93	120.0
C13—N1—Sn1	119.1 (3)	C92—C93—H93	120.0
C17—N1—Sn1	120.1 (3)	C95—C94—C93	119.8 (5)
C23—N2—C27	118.9 (4)	C95—C94—H94	120.1
C23—N2—Sn2	114.9 (3)	C93—C94—H94	120.1
C27—N2—Sn2	125.9 (3)	C94—C95—C96	120.2 (5)
C11—O11—Sn1	129.7 (2)	C94—C95—H95	119.9
C19—O12—Sn1	132.0 (3)	C96—C95—H95	119.9
C21—O21—Sn2	126.6 (2)	C91—C96—C95	120.9 (4)
C29—O22—Sn4	120.8 (2)	C91—C96—H96	119.6
O11—C11—C41	110.5 (3)	C95—C96—H96	119.6
O11—C11—C31	105.7 (3)	C106—C101—C102	118.8 (4)
C41—C11—C31	110.1 (4)	C106—C101—C29	121.2 (4)
O11—C11—C12	110.8 (3)	C102—C101—C29	119.8 (4)
C41—C11—C12	107.5 (4)	C101—C102—C103	120.8 (5)
C31—C11—C12	112.3 (3)	C101—C102—H102	119.6
C13—C12—C11	111.4 (4)	C103—C102—H102	119.6
C13—C12—H12A	109.4	C104—C103—C102	120.1 (5)
C11—C12—H12A	109.4	C104—C103—H103	119.9
C13—C12—H12B	109.4	C102—C103—H103	119.9
C11—C12—H12B	109.4	C103—C104—C105	119.9 (5)
H12A—C12—H12B	108.0	C103—C104—H104	120.1
N1—C13—C14	121.1 (4)	C105—C104—H104	120.1
N1—C13—C12	116.2 (4)	C104—C105—C106	119.9 (5)
C14—C13—C12	122.6 (4)	C104—C105—H105	120.0
C15—C14—C13	118.8 (5)	C106—C105—H105	120.0
C15—C14—H14	120.6	C101—C106—C105	120.5 (5)
C13—C14—H14	120.6	C101—C106—H106	119.8
C14—C15—C16	120.1 (5)	C105—C106—H106	119.8
C14—C15—H15	120.0	C116—C111—C112	118.9 (7)
C16—C15—H15	120.0	C116—C111—C117	121.6 (7)
C15—C16—C17	118.8 (5)	C112—C111—C117	119.4 (7)
C15—C16—H16	120.6	C113—C112—C111	117.0 (7)
C17—C16—H16	120.6	C113—C112—H112	121.5
N1—C17—C16	120.8 (4)	C111—C112—H112	121.5
N1—C17—C18	116.4 (4)	C112—C113—C114	123.7 (7)
C16—C17—C18	122.7 (4)	C112—C113—H113	118.1
C17—C18—C19	116.8 (4)	C114—C113—H113	118.1
C17—C18—H18A	108.1	C115—C114—C113	118.9 (8)
C19—C18—H18A	108.1	C115—C114—H114	120.6
C17—C18—H18B	108.1	C113—C114—H114	120.6
C19—C18—H18B	108.1	C114—C115—C116	120.4 (8)
H18A—C18—H18B	107.3	C114—C115—H115	119.8
O12—C19—C61	108.0 (4)	C116—C115—H115	119.8
O12—C19—C51	110.1 (4)	C111—C116—C115	121.0 (7)
C61—C19—C51	109.7 (4)	C111—C116—H116	119.5
O12—C19—C18	111.0 (3)	C115—C116—H116	119.5
C61—C19—C18	112.0 (4)	C111—C117—H11C	109.5
C51—C19—C18	106.0 (4)	C111—C117—H11B	109.5
O21—C21—C71	106.6 (3)	H11C—C117—H11B	109.5
O21—C21—C81	111.7 (3)	C111—C117—H11A	109.5
C71—C21—C81	110.4 (3)	H11C—C117—H11A	109.5
O21—C21—C22	108.7 (3)	H11B—C117—H11A	109.5
C71—C21—C22	112.7 (4)	C122—C121—C126	119.3 (8)
C81—C21—C22	106.8 (3)	C122—C121—C127	119.7 (8)
C23—C22—C21	109.1 (3)	C126—C121—C127	121.0 (8)
C23—C22—H22A	109.9	C123—C122—C121	121.2 (9)
C21—C22—H22A	109.9	C123—C122—H122	119.4
C23—C22—H22B	109.9	C121—C122—H122	119.4
C21—C22—H22B	109.9	C122—C123—C124	121.2 (10)
H22A—C22—H22B	108.3	C122—C123—H123	119.4
N2—C23—C24	121.5 (4)	C124—C123—H123	119.4
N2—C23—C22	119.7 (4)	C125—C124—C123	115.0 (10)
C24—C23—C22	118.2 (4)	C125—C124—H124	122.5
C25—C24—C23	119.9 (4)	C123—C124—H124	122.5
C25—C24—H24	120.1	C126—C125—C124	125.7 (10)
C23—C24—H24	120.1	C126—C125—H125	117.1
C26—C25—C24	118.1 (5)	C124—C125—H125	117.1
C26—C25—H25	120.9	C125—C126—C121	117.5 (9)
C24—C25—H25	120.9	C125—C126—H126	121.2
C25—C26—C27	121.2 (5)	C121—C126—H126	121.2
C25—C26—H26	119.4	C121—C127—H12C	109.5
C27—C26—H26	119.4	C121—C127—H12D	109.5
N2—C27—C26	119.9 (4)	H12C—C127—H12D	109.5
N2—C27—C28	120.1 (4)	C121—C127—H12E	109.5
C26—C27—C28	119.9 (4)	H12C—C127—H12E	109.5
C27—C28—C29	111.5 (4)	H12D—C127—H12E	109.5
C27—C28—H28A	109.3	C136—C131—C132	121.6 (10)
C29—C28—H28A	109.3	C136—C131—C137	117.3 (9)
C27—C28—H28B	109.3	C132—C131—C137	121.0 (9)
C29—C28—H28B	109.3	C133—C132—C131	117.0 (10)
H28A—C28—H28B	108.0	C133—C132—H132	121.5
O22—C29—C91	110.1 (3)	C131—C132—H132	121.5
O22—C29—C101	111.5 (4)	C134—C133—C132	122.9 (10)
C91—C29—C101	109.1 (3)	C134—C133—H133	118.6
O22—C29—C28	107.8 (3)	C132—C133—H133	118.6
C91—C29—C28	111.4 (4)	C133—C134—C135	118.2 (10)
C101—C29—C28	106.8 (3)	C133—C134—H134	120.9
C32—C31—C36	117.8 (4)	C135—C134—H134	120.9
C32—C31—C11	124.4 (4)	C134—C135—C136	120.8 (10)
C36—C31—C11	117.4 (4)	C134—C135—H135	119.6
C31—C32—C33	120.6 (4)	C136—C135—H135	119.6
C31—C32—H32	119.7	C131—C136—C135	119.1 (10)
C33—C32—H32	119.7	C131—C136—H136	120.5
C34—C33—C32	120.1 (5)	C135—C136—H136	120.5
C34—C33—H33	120.0	C131—C137—H13A	109.5
C32—C33—H33	120.0	C131—C137—H13B	109.5
C33—C34—C35	120.1 (5)	H13A—C137—H13B	109.5
C33—C34—H34	119.9	C131—C137—H13C	109.5
C35—C34—H34	119.9	H13A—C137—H13C	109.5
C36—C35—C34	119.9 (5)	H13B—C137—H13C	109.5
C36—C35—H35	120.0	C142—C141—C143^ii^	119.3 (6)
C34—C35—H35	120.0	C142—C141—H141	120.3
C35—C36—C31	121.4 (4)	C143^ii^—C141—H141	120.3
C35—C36—H36	119.3	C143—C142—C141	120.8 (6)
C31—C36—H36	119.3	C143—C142—H142	119.6
C46—C41—C42	118.6 (4)	C141—C142—H142	119.6
C46—C41—C11	121.5 (4)	C142—C143—C141^ii^	119.9 (6)
C42—C41—C11	119.8 (4)	C142—C143—H143	120.0
C41—C42—C43	119.6 (5)	C141^ii^—C143—H143	120.0
C41—C42—H42	120.2	C141^ii^—C147—H14A	109.5
C43—C42—H42	120.2	C141^ii^—C147—H14B	109.5
C44—C43—C42	120.6 (6)	H14A—C147—H14B	109.5
C44—C43—H43	119.7	C141^ii^—C147—H14C	109.5
C42—C43—H43	119.7	H14A—C147—H14C	109.5
C45—C44—C43	120.4 (5)	H14B—C147—H14C	109.5

Symmetry codes: (i) −*x*+2, −*y*+2, −*z*; (ii) −*x*+1, −*y*, −*z*+1.

### Hydrogen-bond geometry (Å, º)

**Table:**

*D*—H···*A*	*D*—H	H···*A*	*D*···*A*	*D*—H···*A*
C22—H22*A*···O9	0.99	2.28	3.052 (5)	134
C28—H28*A*···O7	0.99	2.48	3.378 (5)	151
C33—H33···O1^iii^	0.95	2.53	3.205 (6)	128

Symmetry code: (iii) −*x*+1, −*y*+1, −*z*.
