# Supplementary figures and images for: Crystal structure of a mixed-valence μ-oxide Sn12 cluster
Source: Acta Crystallogr Sect E Struct Rep Online. 2014 Oct 29;70(Pt 11):m378–9. doi: 10.1107/S1600536814023460 (PMC4257312; doi:10.1107/S1600536814023460)

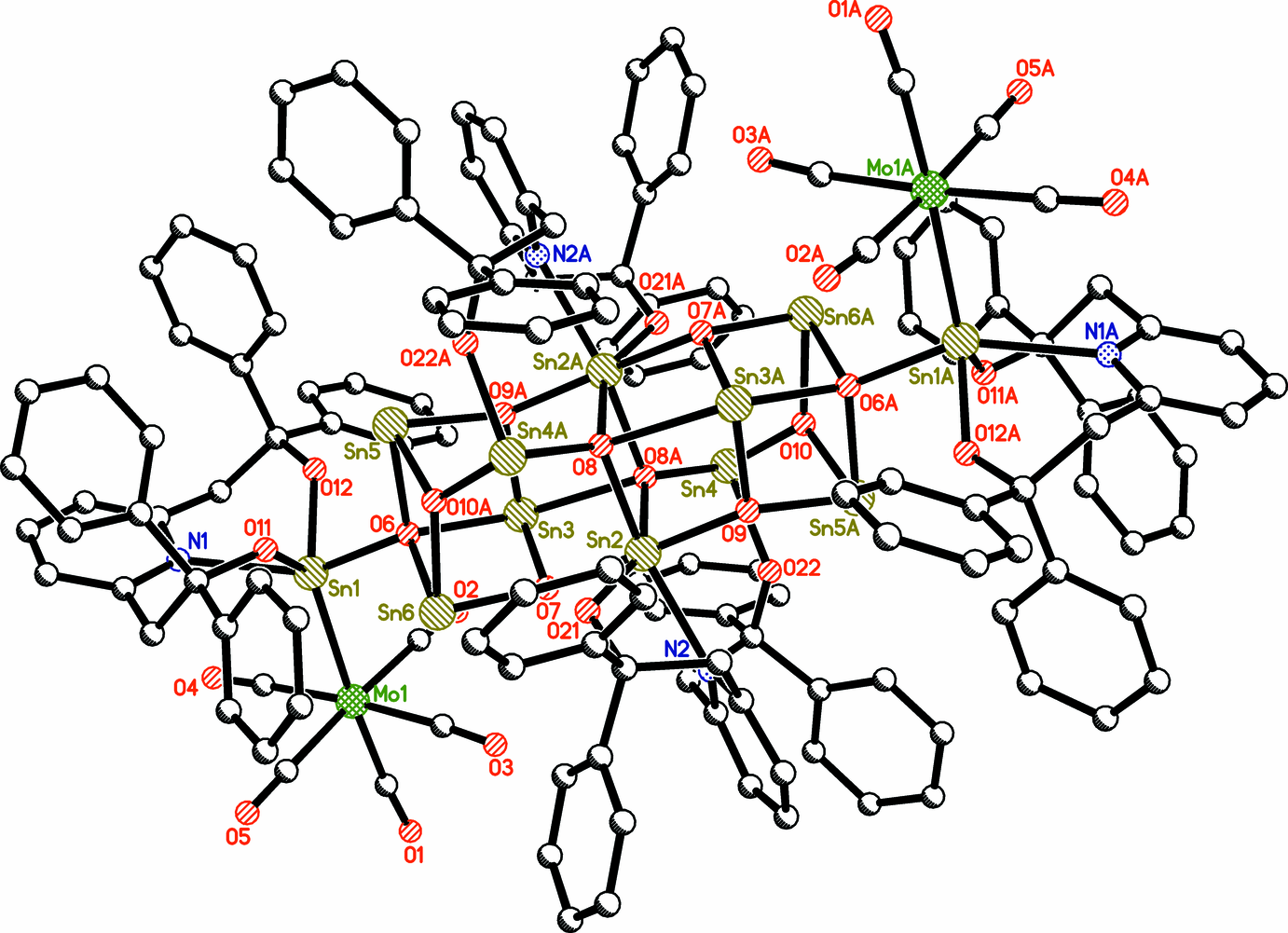

Supplement: Supplementary file 3 [file e-70-0m378-fig1.tif]
